# The Anti-Digestive Characteristics, Effects of Prebiotic Properties on NC and T2DM Mice of *Achyranthes bidentata Polysaccharide*, and the Hypoglycemic Effect of Its Fermentation Products

**DOI:** 10.3390/nu17203249

**Published:** 2025-10-16

**Authors:** Ting Xia, Zhenjie Liu, Wenya Ding, Liting Deng, Xinyang Ning, Jianfang Feng

**Affiliations:** 1 Guangxi Engineering Technology Research Center of Advantage Chinese Patent Drug and Ethnic Drug Development, Guangxi University of Chinese Medicine, Nanning 530200, China; rainman982@126.com (Z.L.); 13878860382@163.com (L.D.); 13879459578@163.com (X.N.); 2 Zhuang Yao Medical Research Institute of Traditional Chinese Medicine, Guangxi University of Chinese Medicine, Nanning 530200, China; 3 School of Traditional Chinese Medicine, Southern Medical University, Guangzhou 510515, China; 4 University Engineering Research Center of Characteristic Traditional Chinese Medicine and Ethnomedicine, Guangxi University of Chinese Medicine, Nanning 530200, China; dingwenya666@163.com; 5 Guangxi Engineering Research Center for High-Value Utilization of Guangxi-Produced Authentic Medicinal Herbs, Nanning 530200, China

**Keywords:** *Achyranthes bidentata polysaccharide*, anti-digestive, prebiotic properties, fermentation

## Abstract

**Background/Objectives**: *Achyranthes bidentata* (AB), recognized as a food and traditional Chinese medicine, exhibits notable biological activity. Our previous study showed the hypoglycemic effect of *Achyrantha bidentata polysaccharide* (ABP). The properties and digestion process of polysaccharides affect their pharmacological activities. The digestion characteristics of ABP are unclear. In this study, we aimed to explore the characteristics of ABP’s simulated digestion and its prebiotic properties and hypoglycemic effects. **Methods**: We used simulated digestion methods to investigate the alterations in ABP levels in the process of digestion and fermentation. Animal experiments were used to compare the effects of the prebiotic properties of ABP on normal control (NC) and type 2 diabetes mellitus (T2DM) mice. Then, in order to further verify the hypoglycemic effect of ABP after fermentation (ABPF), α-glucosidase activity and glucose uptake in Caco-2 cells were examined. **Results**: The results showed that ABP was anti-digestive and mainly degraded by the intestinal flora. Moreover, ABP showed a stronger promoting advantage against beneficial bacteria and inhibited harmful bacteria in the T2DM mice. Compared with NC mice, after ABP treatment, T2DM mice showed a higher increase in levels of short-chain fatty acids (SCFAs). Additionally, the glucose uptake and α-glucosidase activity of Caco-2 cells were significantly decreased after treatment with ABPF. **Conclusions**: These results underscore the potential of ABP as a prebiotic candidate for gut health promotion and T2DM alleviation.

## 1. Introduction

### 1.1. The Pharmacological Relevance of ABP

Achyranthes bidentata (AB), a member of the amaranth family, is traditionally used as both food and medicine. AB nourishes the liver and kidneys and strengthens muscles and bones. Recent studies have shown that AB is rich in polysaccharides, steroids, and saponins that are safe and have strong efficacy. The AB has multiple pharmacological activities, including anti-inflammatory and analgesic effects, lowering of blood pressure, neuroprotection, regulation of immunity, antitumor activity, and lowering of blood sugar levels. Polysaccharides are the main active ingredients in AB, and their biological activities have been widely confirmed. *Achyranthes bidentata polysaccharides* (ABPs) alleviate endoplasmic reticulum stress in osteoarthritic mice by activating the IncRNAEAT1/miR-377-3p signaling pathway [1]. Evidence showed that ABPs mitigate cyclophosphamide-induced side effects by modulating the cytokine balance in helper T cells [2]. The kidney damage in type 2 diabetes mellitus (T2DM) mice can be alleviated by fructan of ABP via modulating the gut microbiota [3]. The anti-osteoporosis effect of ABP has been widely demonstrated [4]. We indicated that ABP alleviates T2DM by regulating intestinal flora and increasing short-chain fatty acid (SCFA) levels [5]. Although ABP has multiple biological activities, its digestion and absorption dynamic process in the body after oral administration is unclear.

### 1.2. The Importance of Digestive Characteristics for Bioactivity

The physical and chemical properties of polysaccharides affect their pharmacological activities [6]. After polysaccharides enter the digestive tract orally, the pH and enzymes in the digestive tract may change the physical and chemical characteristics, affecting their biological activity. Therefore, studying the digestive behavior of polysaccharides is key to exploring their pharmacological activities. Simulations of the digestive environment and digestion time in humans can reflect the oral absorption behavior of polysaccharides, which is simple and has high repeatability [7]. Different polysaccharides undergo different digestion processes. Li et al. showed that *Nostoc commune* Vauch polysaccharides (NCVPs) can be degraded in the digestive tract [8]. By investigating the simulated digestion and fermentation characteristics of ABP, this study provides a theoretical basis for determining its dosage and administration methods as a functional food, thereby establishing a research foundation for the development of ABP-based functional food products. Furthermore, studies have indicated that certain plant polysaccharides exhibit a digestion-resistant nature and exert physiological effects after being fermented by gut microbiota in the colon [9]. Revealing the digestive profile of ABP allows for an in-depth exploration of host–microbiota interactions and lays the groundwork for developing ABP into targeted hypoglycemic therapeutics. Therefore, it is important to explore the digestive characteristics of ABP.

### 1.3. The Role of the Gut Microbiota in Polysaccharide Metabolism

The gut is the second largest organ in the human body and has been shown to be closely related to inflammation, metabolism, and immunity [10]. Intestinal microbial disorders induce various diseases, including intestinal, metabolic, and autoimmune diseases [11]. Intestinal microorganisms possess specific metabolic enzymes that degrade undigested polysaccharides. During digestion, polysaccharides regulate the composition of intestinal bacteria and increase the production of SCFA-producing bacteria [12]. T2DM is closely related to the gut microbiota. A long-term high-sugar, high-fat diet (HSFD) leads to a disorder in the structure of the gut microbiota. Abnormal composition or dysfunctional gut microbiota directly cause metabolic disorders in adipose tissue, muscle, liver, etc. Gut microbiota dysbiosis is closely related to chronic low-grade inflammation caused by obesity [13]. Moreover, harmful substances such as endotoxins in the body are more likely to enter the damaged mechanical barrier of the intestine under inflammatory conditions, leading to more severe intestinal function damage and further gut microbiota dysbiosis; thus, the body enters a vicious cycle, further inducing obesity and T2DM [14].

Our study showed that ABP regulates the gut microbiota and increases SCFAs. However, it is unclear whether ABP exerts its pharmacological effects through intestinal flora fermentation and its regulatory effects on intestinal bacteria in normal control (NC) and T2DM mice. Studying the digestion process of ABP and its effect on intestinal bacteria in NC and T2DM mice is conducive to further elucidating the mechanism by which ABP alleviates T2DM and lays the foundation for the development and research of ABP. However, few studies have simultaneously investigated the effects of ABP or polysaccharides on the intestinal regulation and cell-based hypoglycaemic activity of both normal and diabetic mice.

### 1.4. The Objectives of This Study

In this study, we aimed to investigate the characteristics of ABP during digestion and fermentation, explore its gut microbiota-regulating effects in NC and T2DM mice, and provide a theoretical basis for ABP to alleviate T2DM.

## 2. Materials and Methods

In this study, we used gel permeation chromatography (GPC) and 1-phenyl-3-methyl-5-pyrazolone (PMP) pre-column derivatization to determine the MW (molecular weight) and monosaccharide composition of ABP during the simulated digestion process to clarify its digestion and absorption characteristics. Then, in vitro fermentation and 16S rRNA were used to clarify the intestinal bacteria-regulating effects of ABP, and mice were used to compare the gut microbiota-regulating effects of ABP in NC and T2DM mice. Finally, Caco-2 cells were used to determine the hypoglycemic effects of the fermentation products of ABP in high- and low-glucose media.

### 2.1. Materials and Reagents

The AB was sourced from the Guangdong Province Traditional Chinese Medicine Pieces Factory (Foshan, China); Caco-2 cells were purchased from the National Collection of Authenticated Cell Cultures (Beijing, China); Streptozotocin (STZ), SCFAs were procured from Sigma Company (Munich, Germany); Acarbose (AC), α-amylase, trichloroacetic acid (TCA), analytical-grade ethanol, ethyl acetate, H_2_SO_4_, phenol, 3,5-dinitrosalicylic acid, NaCl, KSCN, KCl, NaHCO3, Na_2_SO_4_, NaH_2_PO_4_, urea, CaCl_2_·2H_2_O, gastric esterase, pepsin, bile salts, and pancreatic enzymes were obtained from Aladdin Biotechnology Co., Ltd. (Shanghai, China); Fetal bovine serum was from Promesa Biotechnology Co., Ltd. (Wuhan, China); DMEM high-glucose medium (glucose concentration of 25 mmol/L), DMEM low glucose medium (glucose concentration of 4 mmol/L), and phosphate buffers were purchased from Gibco Co., Ltd. (Waltham, MA, USA); Cell lysate, α-glucosidase assay kit, glucose content assay kit, and alkaline phosphatase assay kit were purchased from Biyuntian Biotechnology Co., Ltd. (Shanghai, China).

### 2.2. ABP Extraction

The ABP was degreased with ethyl acetate and extracted with distilled water (3 h, twice). TCA (5%) and alpha-amylase were used to remove protein and starch, and ABP was obtained after dialysis, alcohol precipitation, and freeze-drying. Previous studies have shown that ABP has a high purity and low impurity content, meeting the requirements of this study. The MW of the extracted ABP was 41,083 Da; it was composed of Man, Rib, Rha, Glu-UA, Gal-UA, Glu, Gal, Ara, and Fuc. Glu, Gal-UA, and Man were the main monosaccharides identified [5].

### 2.3. In Vitro Digestion

#### 2.3.1. Saliva Digestion

The formula for simulated saliva (pH = 7.0) is presented in Table 1. The ABP digestion in simulated saliva was performed by mixing the polysaccharide solution directly with simulated saliva, as previously reported [15]. Saliva (5 mL) and purified water (15 mL) were added to the ABP solution (8 mg/mL, 20 mL). A shaker at 37 °C and an oscillation frequency of 150 r/min was used to simulate the oral digestion of ABP. Two milliliter mixtures were collected at 0, 0.5, and 1 h and boiled for 5 min. Saliva-digested ABPs (ABP-S) were obtained (*n* = 3).

#### 2.3.2. Gastric Digestion

The simulated gastric formula (pH = 3.0) is shown in Table 1. A saliva-digested ABP sample (20 mL) was added to 20 mL of simulated gastric acid, mixed, and the solution acidity was adjusted to pH 2. The process of gastric digestion was simulated at 37 °C and 150 r/min. Samples were collected at 0, 1, 2, and 4 h and inactivated. Gastric-digested ABPs (ABP-G) were obtained (*n* = 3).

#### 2.3.3. Small Intestine Digestion

The pH of gastric-digested ABP (20 mL) was adjusted to 6.8 and mixed with 20 mL of simulated small intestinal solution (pH = 3.0) (Table 1). The ABPs were digested at 37 °C and 150 r/min, then were collected at 0, 1, 2, and 4 h and inactivated. Small intestine-digested ABPs were obtained (*n* = 3).

### 2.4. In Vitro Fermentation

#### 2.4.1. Fermentation Medium

In vitro fermentation was chosen to closely mimic ABP in the human gastrointestinal tract. The fermentation process of ABP was evaluated through MW, carbohydrate contents, pH, and the content of SCFAs produced during the fermentation process. The fermentation medium was modified from one previously reported [16]. Briefly, mix yeast extract (2.0 g), peptone (2.0 g), NaHCO_3_ (2.0 g), bile salt (0.5 g), NaCl (0.1 g), KH_2_PO_4_ (0.04 g), KHPO_4_ (0.01 g), CaCl_2_ (0.01 g), MgSO_4_ (0.01 g), chlorinated hemoglobin (0.02 g), and Tween 80 (2.0 mL) to prepare 1L of the culture medium. Adjust the pH to 7.0 using HCl and sterilize for 20 min at 121 °C. Then, L-cysteine (0.5 g) and vitamin K1 (10.0 μL) were added to the fermentation medium.

#### 2.4.2. In Vitro Fermentation Experiment

The C57BL/6 mice fecal samples were dissolved in phosphate buffer saline (PBS) to obtain a 20% (*w*/*v*) suspension, vortexed for 5 min, then filtered, and the filtrate was collected. Intestinal bacterial fluid was obtained. Next, the intestinal bacterial fluid was divided into three groups. Each group contained 1 mL of bacterial solution and 9 mL of fermentation culture solution, and then was added with 100 mg of small intestinal-digested ABP (ABP-I group), 100 mg fructooligosaccharides (FOS) (FOS group), and 100 mg PBS (10%) (blank group). The samples were incubated at 37 °C. An anaerobic chamber (the oxygen content: 0.5%) was used to maintain anaerobic conditions throughout the manure fermentation process. The ABP fermentation samples (ABPF) with fermentation times of 0, 2, 4, 6, 12, 24, and 48 h were collected and centrifuged (9000× *g*, 15 min), and the supernatant was stored at −80 °C (*n* = 3).

### 2.5. Animal Experiments

In our previous reports, we demonstrated that ABP has hypoglycemic effects. In this study, an overall animal experiment was used to investigate the effects of ABP on the intestinal flora of normal (NC) mice and T2DM mice, providing a basis for ABP’s role in improving diabetes from the perspective of pathological conditions. Forty-eight C57BL/6J mice, 5–6 weeks old, weighing 22 ± 2 g, were purchased from the Medical Laboratory Animal Center of Guangdong Province (Guangzhou, China). All mice were raised in an SPF barrier environment. The room temperature was 23 ± 1 °C and the relative humidity was 50–60%. Animals within the same group were co-housed (for NC mice, 4 mice per cage, two cages per group; for T2DM mice, 2 mice per cage, four cages per group). They were illuminated and circulated in the dark. All experimental animal protocols were approved by the Southern Medical University Laboratory Animal Ethics Committee. The mice license number is SCXK (Guangdong) 2016-0041.

#### 2.5.1. Effect of ABP on Gut Microbiota in NC Mice

Following a 7-day acclimation period, 24 mice were randomly assigned to three groups using a random number table generated by IBM SPSS 27 software: a blank group of NC mice (NC-Blank group), an ABP group of NC mice (NC-ABP group), and an FOS group of NC mice (NC-FOS group) (*n* = 8). The treatment groups were orally administered with 200 mg/kg ABP and FOS (doses were based on a previous efficacy study). The blank group received an equal dose of physiological saline via oral gavage. After 21 consecutive days of administration, mice were euthanized by dislocating their cervical vertebrae, and the cecal contents of mice were harvested and preserved at −80 °C.

#### 2.5.2. Effect of ABP on Gut Microbiota in T2DM Mice

Development of the T2DM mouse model: After 7-day adaptive feeding, the mice were fed a HSFD consisting of lard, sucrose, cholesterol, sodium deoxycholate, and standard feed (10:20:1:0.2:68.8) for 4 weeks, fasted for 12 h, and allowed free access to water. The STZ was dissolved in 100 mM citrate buffer with a pH range of 4.2–4.5 into a 1% STZ solution. Mice treated with HSFD were injected with 1% STZ solution (50 mg/kg). On the third day after the injection of STZ, fasting blood sugar (FBG) of the mice was determined. When the FBG of mice was ≥11.1 mmoL/L, accompanied by increased urine output, weight loss, and increased water consumption, the model was successfully established. For the remaining mice whose modeling failed, the above procedure was repeated until successful modeling was achieved. Due to the mice’s tolerance to STZ, the number of STZ injections could not exceed three times. The T2DM mice were randomly assigned to three groups using a random number table generated by IBM SPSS 27 (eight mice per group): blank (T2DM-Blank group), ABP (T2DM-ABP group), and FOS (T2DM-FOS group), and administered via oral gavage physiological saline, ABP, and FOS, respectively. The subsequent experimental protocols were performed similar to those mentioned under Section 2.5.1.

### 2.6. Cell Experiments

In order to further verify whether ABP exerts pharmacological effects after fermentation, Caco-2 cells were used to analyze the hypoglycemic effect of ABPF.

#### 2.6.1. Caco-2 Cell Cultures

According to the previous methods [17], Caco-2 cells were divided into two groups and cultured in standard culture medium or DMEM high-glucose medium, and normal Caco-2 cells (NC-Caco-2 cells) and high-glucose Caco-2 cells (HG-Caco-2 cells) were obtained. The cells were isolated and passaged when the cell density reached 90%, and passages with 20–30% of cells were used in the following experiments. Each treatment was independently repeated three times.

#### 2.6.2. Cell Proliferation

The HG-Caco-2 cells were used to investigate the effects of different ABPF concentrations on cell proliferation. The ABPF masterbatches were diluted in the DMEM high-glucose medium to 50, 100, 200, 400, and 800 μg/mL. After HG-Caco-2 adhesion, different concentrations of ABPF culture media (Af) and DMEM high-glucose medium as a control (Ac) were added, and the absorbance was measured at 450 nm after 24 h of incubation. The cell survival rate was considered as 100% after the addition of the DMEM high-glucose medium for 1 h. Cell survival was determined using the following formula: cell viability (%) = Af/Ac.

#### 2.6.3. Effect on Glucose Uptake and of α-Glucosidase Activity

Eight hundred microliters of 28 mmol/L maltose was added as the substrate to each well after cell adhesion. The NC-Caco-2 cells and HG-Caco-2 cells were added to 200 μL of standard medium as control (NC-CON and HG-CON), and the positive group (AC) was added 200 μL of AC solution (25 μg/mL) in the HG-Caco-2 cells. In the experiment groups, 200 μL ABPF solution (the concentration with the highest cell survival rate) was added to the NC-Caco-2 cells and HG-Caco-2 cells (NC-ABPF and HG-ABPF). The cells were lysed at 30, 60, and 120 min after dosing, and the supernatant was centrifuged using a glucose content assay kit to determine the glucose content. Cell lyses were sampled via centrifugation after 120 min of dosing, using the α-glucosidase assay kit to test α-glucosidase activity.

### 2.7. Related Indexes Detection

#### 2.7.1. Physicochemical Properties of ABP

The reducing sugar and carbohydrate contents of ABP were determined using HPLC and UV spectroscopy, respectively [18]. The MW of ABP and its digested samples were measured using GPC; ABP samples and T series dextran standard substances were dissolved in 0.05 M NaCl (5.0 mg/mL). The MW of ABP was calculated by calibrating the chromatographic column using dextran standards of different MWs. The types and proportions of monosaccharides in ABP were tested using PMP combined with HPLC [19]. The standards included Mannose, Ribose, Rhamnose, Glucuronic Acid, Galacturonic Acid, Aal-UA, Glucose, Galactose, Arabinose, and Fucose. Fourier transform infrared (FT-IR) spectroscopy was used to analyze the chemical structure of ABP and its digestion products.

#### 2.7.2. Determination of SCFAs

The SCFA determination method has been outlined in a previous study [20]. Fecal samples (1 g) were fully dissolved in 10 mL of PBS, and the supernatant solution was collected after centrifugation (4 °C, 10 min). The fecal supernatant or fermentation supernatant was mixed with 50% sulfuric acid after vortexing for 2 min, and ether was added to separate the organic layers. 2-ethylbutyric acid was used as the internal standard. The parent and daughter ions of the SCFAs were determined using gas chromatography-mass spectrometry (GC-MS), and the SCFA contents were calculated using the internal standard method.

#### 2.7.3. Gut Microbiota Analysis

Colon contents were carefully collected under sterile conditions and preserved at −80 °C until further use. DNA from fecal samples was extracted using a kit and stored at −80 °C for gene sequencing. The bacterial 16S rRNA V3-V4 region was amplified using forward primer 338F (5′-ACTCCTACGGAGGCAGCAG-3′) and reverse primer 806R (5′-GGACTACHVGGTWTCTAAT-3′). After this process, each sample was sequenced using an Illumina MiSeq platform (Illumina, San Diego, CA, USA). Shanghai Meiji Biotechnology Co., Ltd. (Shanghai, China) performed 16S rRNA sequencing.

### 2.8. Statistical Analysis

Data were shown as mean ± standard (mean ± SD). Analyses were carried out using GraphPad Prism 9.0. Data with 16S rRNA sequencing were analysed by the Wilcoxon rank-sum test, and the other data were analysed by one-way analysis of variance (ANOVA). Post hoc tests were used after ANOVA. Multiple comparisons for 16S rRNA are based on the results of the homogeneity of variance test. If the variances are homogeneous, the LSD method is used; otherwise, the Dunnett’T3 method is employed. Significance between groups was shown as *p* < 0.05 and *p* < 0.01. In this study, the 16S rRNA sequencing in mice was conducted as 6 independent repeated experiments (*n* = 6), while the in vitro digestion, fermentation, and cell experiments were carried out as 3 independent repeated experiments (*n* = 3).

## 3. Results

### 3.1. In Vitro Digestion of ABP

#### 3.1.1. Saliva Digestion

**Figure 1:**
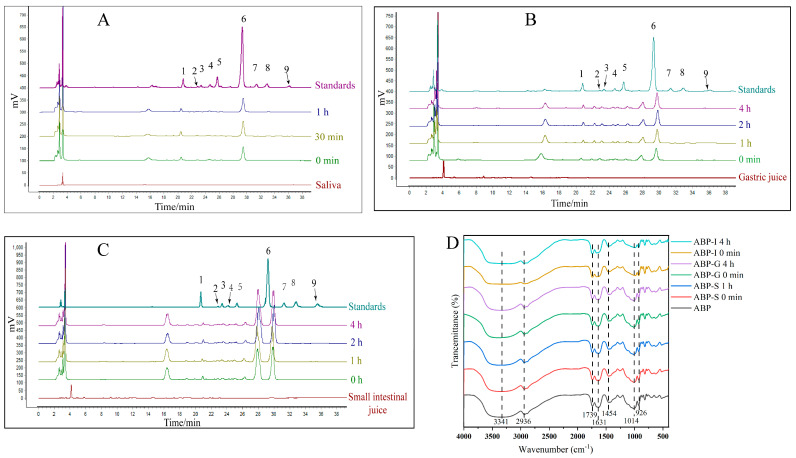
Monosaccharides and FI-IR of ABP. Monosaccharides in the simulated saliva digestion (**A**); simulated gastric juice digestion (**B**); simulated small intestinal juice digestion (**C**); FI-IR in the simulated digestion process (**D**). (1. Mannose; 2. Ribose; 3. Rhamnose; 4. Glucuronic Acid; Galacturonic Acid; 5. Aal-UA; 6. Glucose; 7. Galactose; 8. Arabinose; 9. Fucose).

Salivary amylase is the principal enzyme responsible for digestion in the oral cavity, with α-amylase catalyzing the hydrolysis of starch into smaller saccharides. After 1 h of simulated salivary digestion, the MW of ABP decreased from 41,083 Da to 40,662 Da, representing a 0.9% reduction (*p* < 0.05). Concurrently, reducing sugar levels within the digestive solution exhibited a significant rise, increasing from 0.08 mg/mL to 0.13 mg/mL (*p* < 0.05) (Table 2). Analysis of the monosaccharide composition revealed that no free monosaccharides were detected during the in vitro simulated oral digestion of ABP (Figure 1A).

FT-IR results showed that ABP and ABP-S displayed similar spectral absorption characteristics (Figure 1D). A prominent and broad band was observed at 3341 cm^−1^, along with a weaker absorption peak at 2936 cm^−1^, which are attributed to the stretching vibrations of O-H and C-H in the -CH_2_ group, respectively. The C-O stretching vibration appeared at 1739 cm^−1^. Peaks at 1631 cm^−1^ and 1454 cm^−1^ were linked to the asymmetric stretching vibration of C-O and the bending vibration of C-H, respectively, suggesting the presence of uronic acid in the ABP. A peak around 1014 cm^−1^ likely indicates C-O-C linkages, while the faint absorption near 926 cm^−1^ might be related to glycosidic bonds in various configurations. The similar FT-IR spectra of ABP and ABP-S suggested that the functional groups of ABP remained unchanged during the simulated oral digestion process. These results indicate that ABP was undigested by saliva. Although slight changes in MW and reducing sugar content were observed, these may result from limited hydrolysis of glycosidic bonds by salivary α-amylase. Nevertheless, the overall structure of ABP remains largely unaffected during salivary digestion.

#### 3.1.2. Gastric Digestion

Following a 4 h period of simulated gastric digestion, the MW of ABP showed a reduction from 40,642 Da to 39,562 Da, indicating a 2.7% decline (*p* < 0.05). Concurrently, the amount of reducing sugars increased from 0.13 mg/mL to 0.18 mg/mL (*p* < 0.05) (Table 2). Nevertheless, the HPLC analysis of the monosaccharide composition indicated the absence of free monosaccharides throughout the simulated gastric digestion (Figure 1B). The FT-IR results demonstrated that ABP, ABP-S, and ABP-G exhibited similar absorption spectra (Figure 1D), suggesting that there were no changes in the structural characteristics of ABP during the simulated gastric digestion process. These findings indicate that ABP was not digested by the gastric mucosa. The changes in MW and reducing sugar content observed during gastric simulation were probably a result of the disruption of polysaccharide chains in the acidic environment of the stomach.

#### 3.1.3. Small Intestine Digestion

There were no notable alterations in the MW, reducing sugar content, monosaccharide composition, or FT-IR characteristics of ABP during intestinal digestion (*p* > 0.05), as shown in Table 2, Figure 1C,D. These findings indicate that ABP is resistant to enzymatic breakdown under conditions that simulate the small intestine.

### 3.2. In Vitro Fermentation of ABP

#### 3.2.1. Relative MW and Total Carbohydrate Contents

**Figure 2:**
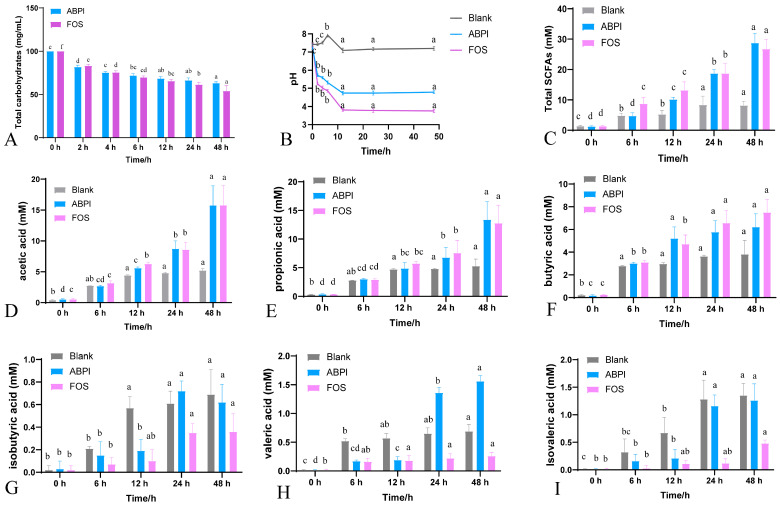
Dynamics changes of ABP during in vitro fermentation (*n* = 3). Residual carbohydrate (**A**); pH (**B**); total SCFAs (**C**); acetic acid (**D**); propionic acid (**E**); butyric acid (**F**); isobutyric acid (**G**); valeric acid (**H**); and isovaleric acid (**I**). Different letters indicate a significant difference at *p* < 0.05.

During in vitro fecal fermentation following simulated small intestinal digestion, the MW and residual carbohydrates of ABP were monitored. A marked reduction in MW was observed from 39,573 to 10,983 Da over 24 h. This decline was more pronounced during the first 6 h and slowed thereafter. In parallel, the total carbohydrate contents of ABP and FOS decreased to 36.9% and 45.9% of their initial values, respectively, with most of the degradation occurring within the first 6 h of fermentation (Table 3 and Figure 2A). These results indicate that both ABP and FOS serve as fermentable carbon sources, which are subjected to microbial degradation and utilized by the gut microbiota.

#### 3.2.2. pH and SCFA Contents

Earlier research demonstrated that the gut microbiota can break down dietary fiber to produce SCFAs, which subsequently decrease the pH of the gut environment. As shown in Figure 2B, all experimental groups exhibited a decreasing trend in pH during fermentation. Compared to the blank group, the pH decrease was more pronounced in the ABP and FOS groups, with ABP leading to a lower pH than FOS throughout the fermentation period.

Similar to most plant-derived polysaccharides, ABP resists digestion in the human gastrointestinal tract. It is fermentable by the gut microbiota and produces SCFAs during fermentation. In our study, the SCFA concentrations increased with increasing fermentation time. After 48 h, total SCFA concentrations rose from 1.43 ± 0.21 mM to 8.20 ± 1.37 mM in the blank group, from 1.29 ± 0.17 mM to 28.75 ± 3.16 mM in the ABP group, and from 1.37 ± 0.26 mM to 26.76 ± 3.16 mM in the FOS group (Figure 2C). These results revealed that the fermentation of ABP and FOS significantly enhanced SCFA production compared to that of the blank control. Regarding individual SCFAs, no notable differences were observed in the levels of acetic, propionic, or butyric acids between the ABP and FOS groups. However, the ABP group exhibited significantly higher concentrations of isobutyric acid, valeric acid, and isovaleric acid than the FOS group (Figure 2D–I).

### 3.3. The Effects of ABP on Regulating Gut Microbiota

#### 3.3.1. The Effect of ABP on the Gut Microbiota of NC Mice

**Figure 3:**
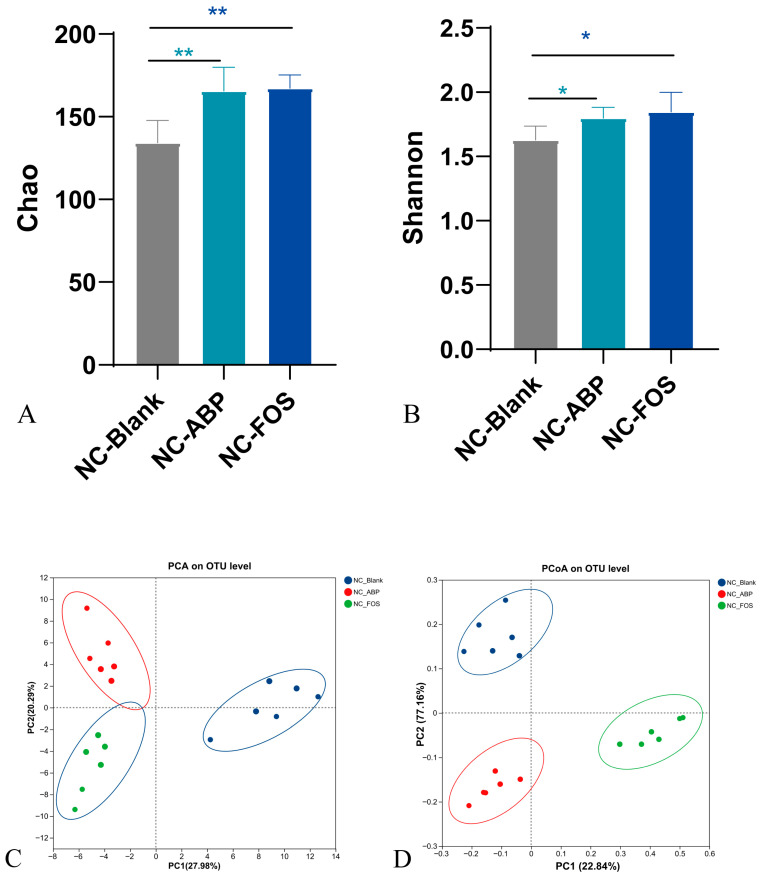

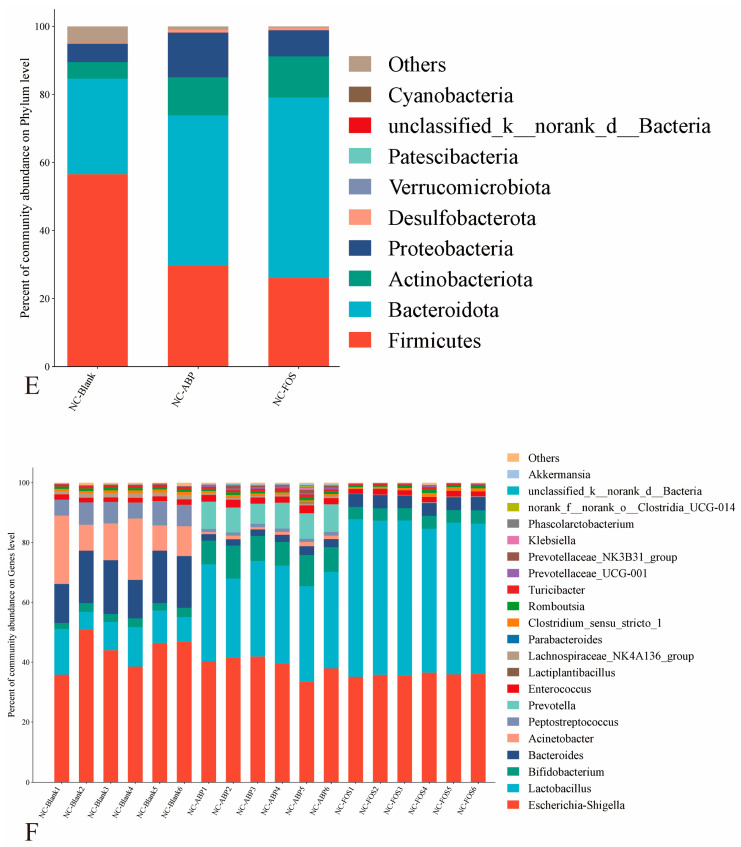

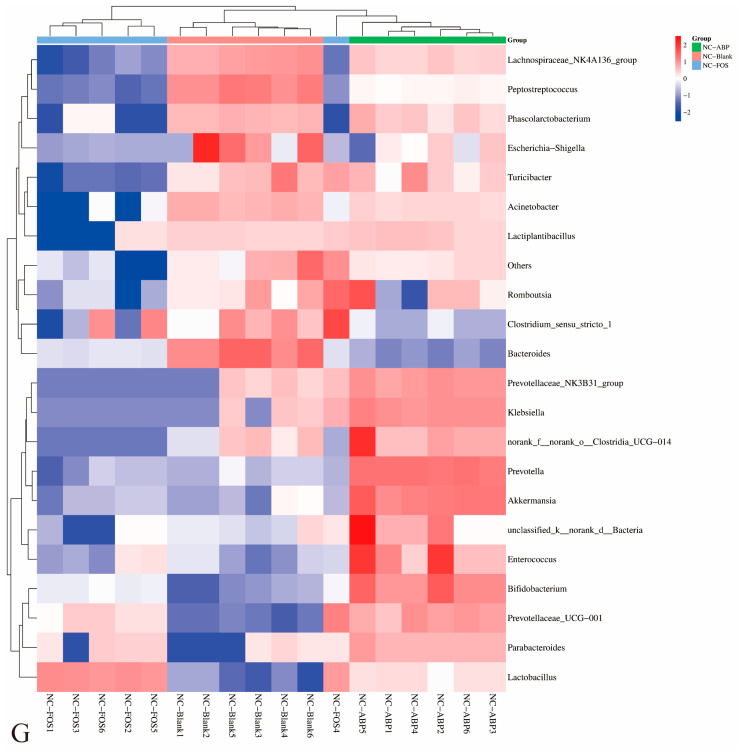

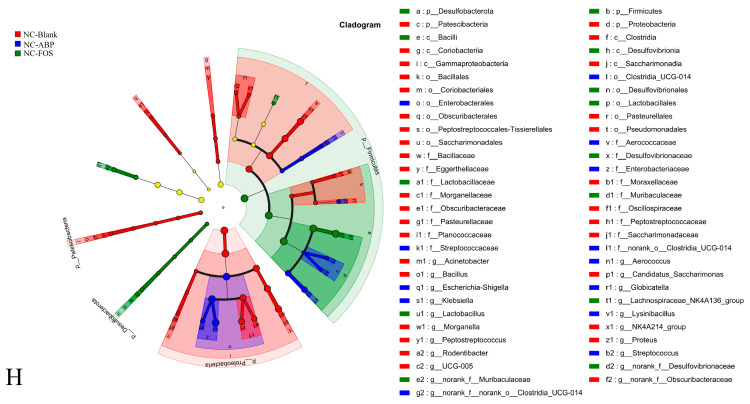
16S rRNA analysis of NC mice (*n* = 6). Chao (**A**) and Shannon (**B**); PCA (**C**) and PCoA (**D**); analyzed at the phylum level (**E**) and genus level (**F**); Heatmap of the top 20 bacterial genera (**G**); LEfSe analysis at the gene level (**H**). * *p* < 0.05, ** *p* < 0.01.

A total of 1,171,570 optimized sequences were obtained, covering 500,819,370 bp. The Sobs index was used as a diversity metric, and the rarefaction curve gradually leveled off, confirming the reliability of the sequencing approach (Figure S1). The α-diversity analysis, including Chao and Shannon, showed that after 21 days of ABP treatment, the diversity of intestinal bacteria in the NC-ABP and NC-FOS groups was significantly higher than that in the NC-Blank group (Figure 3A,B). The PCA and principal coordinates analysis (PCoA) were used to analyze the microbial communities among the groups. Our findings revealed that the microflora of the NC-ABP and NC-FOS groups were closely clustered, whereas they were clearly separated from the NC-Blank group (Figure 3C,D), suggesting that ABP and FOS increased the diversity of the microbiota in NC mice.

At the phylum level, oral administration of ABP notably decreased *Firmicutes* abundance (*p* < 0.05) and enhanced *Bacteroidota*, *Actinobacteriota*, and *Proteobacteria* abundances (*p* < 0.05), mirroring the alterations observed in the NC-FOS group (Figure 3E). At the genus level, both ABP and FOS treatments significantly increased the relative abundance of beneficial genera, including *Lactobacillus*, *Bifidobacterium*, *Prevotella*, *Prevotellaceae_UCG-001*, *Prevotellaceae_NK3B31_group*, and *Akkermansia*, and decreased the abundance of harmful genera, including *Acinetobacter*, *Lachnospiraceae_NK4A136_group*, and *Phascolarctobacterium* (Figure 3F).

These results indicate that ABP and FOS act as prebiotics in NC mice, facilitating the proliferation of advantageous bacterial populations. Variations in microbial communities among the groups are illustrated in the heatmap presented in Figure 3G. A visualization of the LEfSe analysis is shown in Figure 3H.

#### 3.3.2. The Effect of ABP on the Gut Microbiota of T2DM Mice

**Figure 4:**
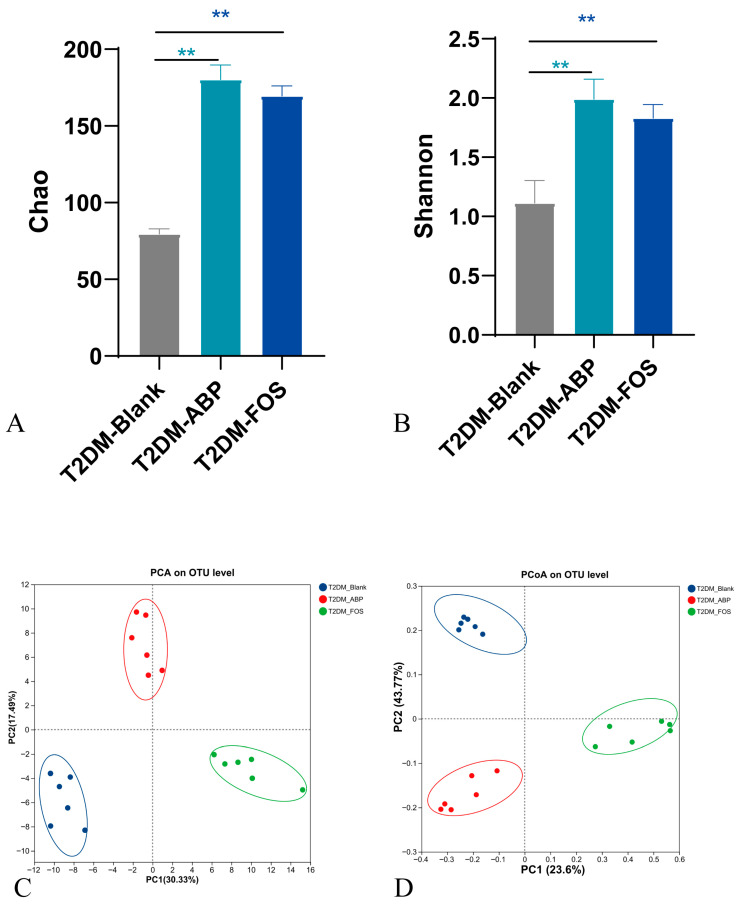

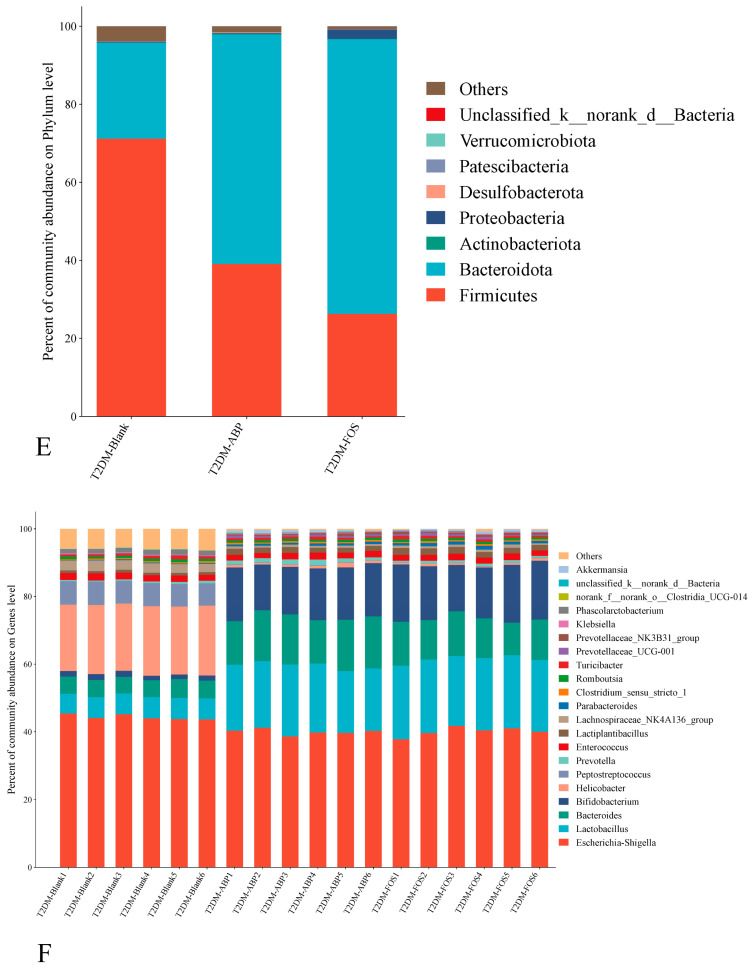

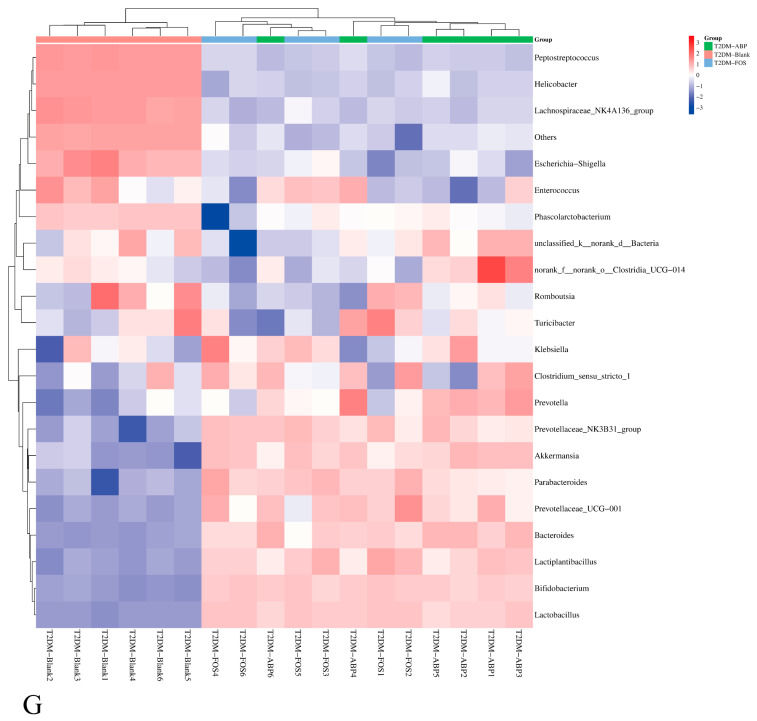

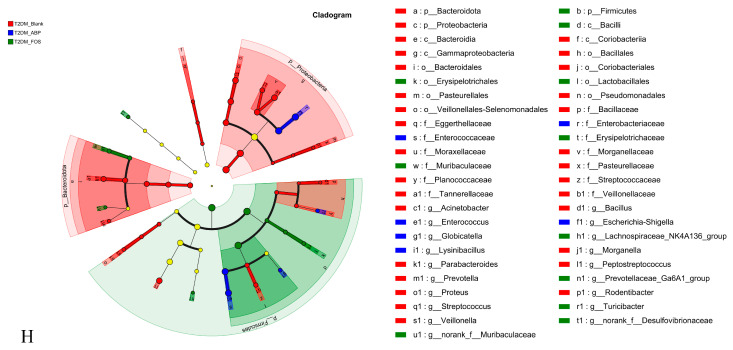
16S rRNA analysis of T2DM mice (*n* = 6). Chao (**A**) and Shannon (**B**); PCA (**C**) and PCoA (**D**); analyzed at the phylum level (**E**) and genus level (**F**); Heatmap of the top 20 bacterial genera (**G**); LEfSe analysis at the gene level (**H**). ** *p* < 0.01.

The T2DM mice were orally administered ABP, FOS, or physiological saline for 21 consecutive days. The Sobs index of 16S rRNA is shown in Figure S2. Gut microbiota analysis revealed that both ABP and FOS treatments significantly increased α-diversity in T2DM mice, similar to the effects observed in NC mice. The Chao and Shannon index analyses further confirmed an increase in bacterial diversity in the T2DM-ABP and T2DM-FOS groups (Figure 4A,B). The PCA and PCoA results showed distinct clustering between the T2DM-Blank and T2DM-ABP groups, with minimal differences between the T2DM-FOS and T2DM-ABP groups (Figure 4C,D). These results indicate that ABP and FOS treatments significantly modulated the gut microbiota of T2DM mice.

The ABP treatment significantly reduced the abundance of *Firmicutes* (*p* < 0.05) and increased that of *Bacteroidetes* (Figure 4E). Furthermore, the F/B ratio was reduced in T2DM mice after ABP treatment, potentially contributing to the alleviation of T2DM symptoms (Figure S3). In addition, ABP significantly elevated the abundance of *Lactobacillus*, *Bacteroides*, *Bifidobacterium*, *Prevotella*, *Prevotellaceae_UCG-001*, *Prevotellaceae_NK3B31_group*, and *Akkermansia* at the genus level. In contrast, it suppressed the levels of potentially harmful genera, including *Helicobacter*, *Peptostreptococcus*, *Lachnospiraceae_NK4A136_group*, and *Phascolarctobacterium* (Figure 4F).

These findings indicate that ABP modulates gut microbiota composition in T2DM mice, fostering the proliferation of beneficial bacteria. A heat map of the bacteria is shown in Figure 4G. The results of LEfSe analyses are shown in Figure 4H.

#### 3.3.3. Comparison of the Effects of ABP on Gut Microbiota and SCFAs in NC and T2DM Mice

**Figure 5:**
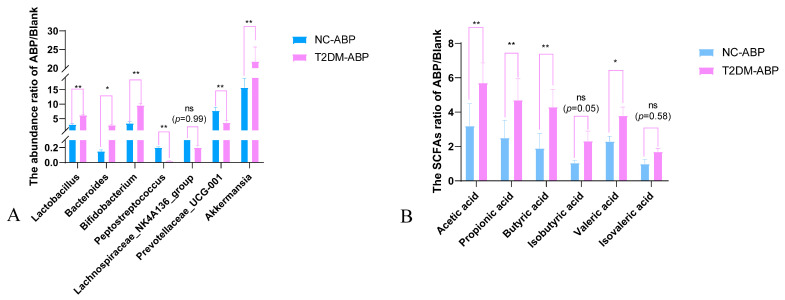
Comparison of the effects of ABP on gut microbiota (**A**) and SCFAs (**B**) in NC and T2DM mice. * *p* < 0.05, ** *p* < 0.01, ns indicates *p* ≥ 0.05.

The ABP functions as a prebiotic that influences the gut microbiota in NC and T2DM mice by enhancing advantageous bacteria and reducing detrimental bacteria. However, the magnitudes of these effects differed. When the microbes of NC and T2DM mice influenced by ABP were elevated, the beneficial bacteria in T2DM mice were higher than those in NC mice. Upon oral ABP intake, the abundance of *Lactobacillus* and *Bifidobacterium* in T2DM mice increased by approximately 2 times and 2.8 times, respectively, compared to that in NC mice. The reduction in the number of harmful bacteria, including *Peptostreptococcus*, *Lachnospiraceae_NK4A136_group*, and *Helicobacter*, was even more significant (Figure 5A). SCFA analysis revealed that ABP significantly increased fecal SCFA levels in T2DM mice compared to those in NC mice (Figure 5B). This effect was most evident for acetic, propionic, butyric, and valeric acids, which were produced by *Lactobacillus*, *Bifidobacterium*, and *Akkermansia*. These findings indicate that under T2DM conditions, the gut microbiota exhibits an improved ability to metabolize ABP, which could hold therapeutic potential for managing T2DM.

### 3.4. Hypoglycemic Effect of ABPF on Caco-2 Cells

#### 3.4.1. Effect of ABPF on Cell Viability

**Figure 6:**
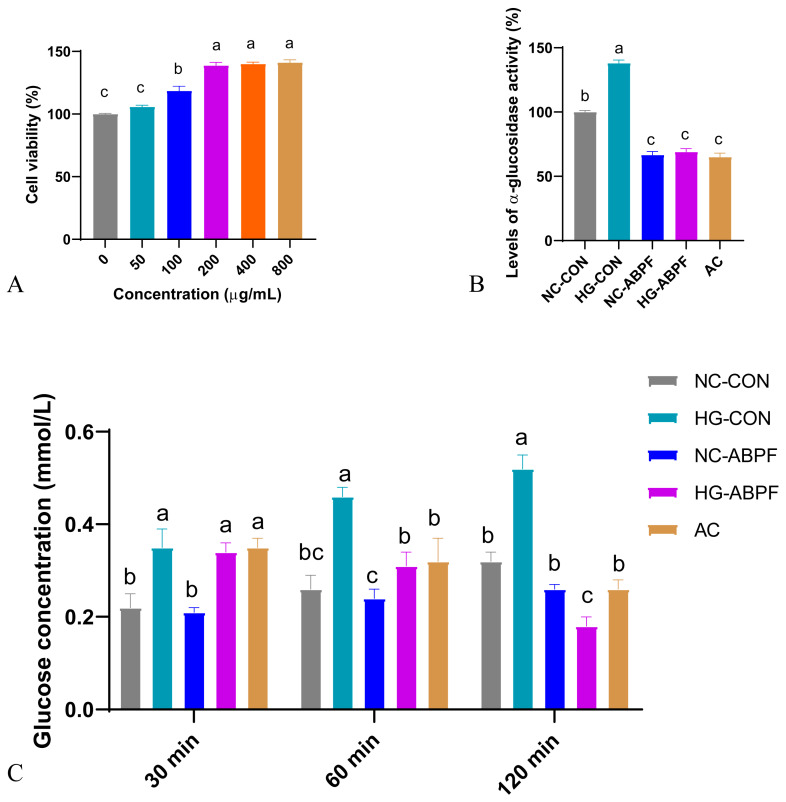
Effect of ABPF on cell viability and glucose uptake in Caco-2 cells (*n* = 3). Caco-2 cells viability (**A**); α-glucosidase activity (**B**); Glucose uptake (**C**). Different letters indicate a significant difference at *p* < 0.05.

It had a proliferative effect on Caco-2 cells within the range of 50 to 800 μg/mL of ABPF (Figure 6A). Compared with the control group, when the concentration of ABPF was 200 μg/mL, the cell survival was 130.8% (*p* < 0.05). The cell survival was not significantly different when the concentration of ABPF rose from 200 to 800 μg/mL. Therefore, we selected 200 μg/mL of ABPF in the subsequent experiments.

#### 3.4.2. Effect of ABPF on Glucose Uptake and α-Glucosidase Activity

Inhibition of α-glucosidase activity can prevent a sharp increase in blood sugar levels after meals, which helps to alleviate T2DM. The ABPF inhibited α-glucosidase activity in both NC and HG-Caco-2 cells; however, the α-glucosidase inhibitory effect of ABPF on HG-Caco-2 cells was stronger than that on NC-Caco-2 cells (Figure 6B). Specifically, in NC-Caco-2 cells, after the addition of ABPF, the inhibitory effects on α-glucosidase activity were 1.5-fold greater than those in the control group, while under the HG-Caco-2 cells conditions, the inhibitory effect of ABPF on α-glucosidase activity was 2.0-fold greater than that in the control group.

No significant differences were observed at 30 min in any of the groups. From 60 min to 120 min, the inhibitory effect of ABPF on the glucose uptake capacity of both NC- and HG-Caco-2 cells increased with time (Figure 6C). Similar to the α-glucosidase activity, the glucose uptake inhibition effect was more effective under the HG-Caco-2 cells condition, which was 2.3-fold higher than the NC-Caco-2 cells. Furthermore, the inhibitory effect of ABPF on glucose uptake by HG-Caco-2 cells was greater than that of AC at 120 min.

## 4. Discussion

In this study, the digestive characteristics of ABP and their effects on the gut microbiota of NC and T2DM mice were investigated using in vitro simulated digestion, fecal fermentation, cell hypoglycemia, and in vivo mouse experiments. Our results indicate that ABP remains undigested in simulated saliva, gastric fluid, and small intestinal fluid but is instead utilized by the gut microbiota, which modulates the microbial community structure and produces SCFAs. Previous reports have shown that most plant polysaccharides are anti-digestible, further supporting our findings. Moreover, our results showed that compared to NC mice, ABP significantly promoted beneficial bacteria and increased SCFA production in T2DM mice, with effects superior to those observed in the control group treated with FOS. Also, compared with the NC-Caco-2 cells, ABPF showed a stronger inhibitory effect on glucose uptake and α-glucosidase in HG-Caco-2 cells.

### 4.1. Digestive Resistance of ABP

The simulated digestion results revealed that saliva caused a slight change in the MW and reducing sugars of ABP, whereas no free monosaccharides were generated. It is inferred that ABP is not digested by saliva. The changes in the relative MW and reducing sugar content were likely due to the hydrolysis of glycosidic bonds in ABP by α-amylase in saliva. This result is consistent with previous findings reported [15]. During gastric digestion, ABP exhibit digestive characteristics similar to those observed during the oral phase, although the mechanisms differ. This is likely due to the sensitivity of polysaccharides to acidic and pH conditions, where the strong acidity of the gastric fluid causes disruption of the molecular chain, resulting in a slight decrease in MW and a slight increase in reducing sugars. Alternatively, contact between the polysaccharide and gastric acid may destroy the initially formed polymer, resulting in a decrease in the relative MW [21]. The MW, total carbohydrates, and free monosaccharides of ABP remained unchanged during the simulated process, suggesting that ABP was undigested in the small intestine. These findings are consistent with those of previous studies. Li [22] evaluated the dynamic digestion process of tamarind seed polysaccharides within the stomach and small intestine and found that the MW of tamarind seed polysaccharides decreased and the reducing sugar content increased, whereas the polysaccharides were not digested by digestive fluids. In summary, most polysaccharides, after in vitro simulated digestion, undergo glycosidic bond cleavage, leading to a decline in MW accompanied by an increase in reducing sugar levels; however, they do not generate free monosaccharides and are not digested by digestive fluids. They require further breakdown and utilization by gut bacteria.

### 4.2. Microbial Fermentation and SCFAs

The fermentation results indicated that both MW and total carbohydrate content of ABP decreased significantly during fermentation. The total carbohydrate content reflects the extent to which ABP is utilized by microorganisms, suggesting that ABP is degraded and metabolized. The observed decline in pH, coupled with an increase in SCFA concentrations in the fermentation broth, further supports the hypothesis that ABP is enzymatically broken down by specific SCFA-producing microbes. These findings were consistent with previous research [23]. pH is involved in intestinal homeostasis, and low pH can inhibit pathogenic bacteria growth to help maintain intestinal homeostasis. The ABP and FOS groups exhibited significantly lower pH values than the blank group, suggesting that ABP and FOS improved the intestinal environment. This effect may represent one of the mechanisms by which ABP exerts its pharmacological activity by modulating the gut microbiota [5]. SCFAs, metabolites produced through the fermentation of ABP, play essential roles in maintaining microbial balance and regulating host energy metabolism [24]. Our findings indicated that SCFAs mainly contain acetic, propionic, and butyric acids. Furthermore, the concentrations of isobutyric, valeric, and isovaleric acids were significantly higher in the ABP group. This variation may be attributed to differences in the metabolic capacities of specific gut microbes that degrade polysaccharides and produce distinct SCFA profiles [25].

SCFAs are a primary energy source for intestinal cells and perform a variety of physiological functions, including the regulation of metabolism, immune response, and oxidative stress [26]. Researchers have confirmed that acetic acid enhances energy metabolism, increases thermogenesis, and regulates glucose and lipid metabolism [27]. Propionic acid enhances gut barrier function and reduces inflammation in both the intestine and liver [28]. Butyric acid has also been implicated in the amelioration of metabolism [29]. In our study, SCFA concentrations increased significantly after 48 h of ABP fermentation, providing mechanistic support for the pharmacological activity of ABP.

### 4.3. Gut Microbiota Modulation

The balance of gut microbiota is strongly associated with metabolism, the immune system, and overall health [30]. Gut microbiota analysis revealed that ABP decreased the F/B ratio in T2DM mice. *Bacteroidetes* possess specific polysaccharide utilization loci that encode enzymes responsible for polysaccharide degradation to provide energy. *Bacteroidetes* primarily break down polysaccharides into oligosaccharide fragments and produce SCFAs [31]. Our results suggest that *Bacteroidetes* may be a key phylum involved in ABP degradation. In the NC mice, ABP treatment increased the *Proteobacteria* abundance. *Actinobacteria* are one of the predominant phyla in the colon and harbor enzymes capable of degrading complex polysaccharides [32,33]. The ABP markedly enhanced the relative abundance of beneficial gut microbes in both NC and T2DM mice, while decreasing the relative prevalence. Further analysis revealed that, compared to NC mice, ABP exhibited a stronger influence in elevating the relative abundance of advantageous bacteria in T2DM mice. Similarly, a more substantial decrease was observed in detrimental bacteria. Moreover, following ABP treatment, T2DM mice displayed notably elevated acetic acid, propionic acid, butyric acid, and valeric acid levels compared to NC mice. These results indicated that T2DM accelerates the degradation of ABP and modulates the gut microbiota by fostering beneficial bacterial proliferation, suppressing harmful strains, and enhancing SCFA levels, thereby contributing to the antidiabetic properties of ABP.

### 4.4. Comparison Between NC and T2DM Mice

Under normal physiological conditions, polysaccharides play a fundamental regulatory role in maintaining gut microbiota diversity and metabolic homeostasis [34]. They consistently produce SCFAs, maintain the pH balance of the intestinal microenvironment, prevent the proliferation of pathogenic bacteria, and maintain the physiological functions of the intestinal barrier. Furthermore, the oral administration of ABP under physiological conditions helps sustain immune homeostasis, promotes balanced differentiation of immune cells, and maintains baseline immune activity. However, in the pathophysiological state of T2DM, the regulatory effects of polysaccharides are complex. They may rectify the imbalance in T2DM mice by increasing the number of beneficial bacteria and decreasing the number of harmful microbes. This aligns with our results showing that ABP boosts *Lactobacillus*, *Bacteroides*, *Bifidobacterium*, and *Akkermansia* abundance in T2DM mice. Furthermore, after treatment with ABPF, the differences in glucose uptake and α-glucosidase activity in Caco-2 cells under normal and high-glucose conditions provided results for mice. Studies have shown that *Lactobacillus* modulates the MMP9 and NOTCH 1 pathways through its interaction with bile acids, thereby improving FBG levels and insulin resistance [35]. In addition, *Lactobacillus* has been widely used as a prebiotic to improve blood glucose [36]. *Bacteroides* ameliorate T2DM by regulating bile acid and lipid metabolism through the gut/liver axis [37]. *Bifidobacterium* is related to insulin resistance and the host [38]. *Akkermansia* enhances glucose metabolism [39]. In our earlier study, we demonstrated that ABP significantly decreased *Peptostreptococcus* and *Lachnospiraceae_NK4A136_group* abundance, which can alleviate T2DM [5]. Compared to the NC mice, ABP significantly increased the levels of SCFAs. As metabolic byproducts of the gut microbiota, SCFAs act not only as an energy source for the microbiota in pathological states but also influence immune cell activity by activating GPRs, thereby contributing to the antidiabetic effects of ABP. Previous studies corroborated this finding [40]. Therefore, the differences in gut microbiota modulation by ABP between the NC and T2DM mice may be attributed to the initial disparity in their microbiota composition. Under NC conditions, the microbiota structure is stable, with polysaccharides primarily playing a role in its maintenance. In contrast, T2DM mice exhibit severe dysbiosis, such as an abnormal increase in the F/B ratio, which can be reversed by polysaccharide treatment [41]. Furthermore, while SCFAs predominantly support energy metabolism in NC mice, SCFAs produced in T2DM mice primarily exert anti-inflammatory and signaling regulatory effects.

### 4.5. Implications for Glucose Metabolism

ABPF inhibited the glucose uptake rate and α-glycosidase activity of Caco-2 cells. Furthermore, this inhibitory effect was more pronounced in HG-Caco-2 cells. The above results indicate that ABP can be developed into a hypoglycemic functional food or health food.

In this study, through detailed SCFA analysis and integration with Caco-2 glucose uptake, we revealed that ABP exhibited a stronger prebiotic effect in T2DM mice, which is a difference from previous studies. However, our research has some limitations. The in vitro simulated digestion cannot fully replicate the in vivo environment, the mice microbiota may differ from that of humans, and the sample size of mice was small; thus, the above results still require further investigation. In the future, we will conduct human clinical studies on the long-term effects of ABP and its interaction with diet or drugs, and investigate the mechanistic pathways linking SCFAs to metabolic improvements to strengthen the scientific rigour of the study.

## 5. Conclusions

In this study, we found that ABP exhibited minimal changes in total carbohydrates, reducing sugars, MW, monosaccharide content, and structural characteristics during simulated gastrointestinal digestion, indicating its anti-digestive properties. In vitro fecal fermentation experiments showed that ABP was primarily degraded by the gut microbiota.

The mouse model experiments revealed that ABP improved the gut microbiota structure in both NC and T2DM mice, promoting the growth of beneficial bacteria while suppressing the expansion of harmful bacteria and increasing SCFA levels. The effects were more significant in the T2DM mice. Moreover, ABP significantly elevates acetic acid, propionic acid, butyric acid, and valeric acid levels in T2DM mice. In addition, ABPF showed a stronger inhibitory effect on glucose uptake and α-glucosidase activity in HG-Caco-2 cells than in NC-Caco-2 cells.

In this study, we employed a combined approach of in vivo, in vitro, and cell-based methods to reveal the differential effects of ABP in T2DM and NC mice. Our results provide initial insights into the digestive process of ABP, underscore its potential to modulate the gut microbiota, and provide a scientific foundation for its use in managing T2DM, which will contribute to the continued development and application of ABP.

## Figures and Tables

**Table 1:** Formulations of salivary, gastric, and small intestinal electrolyte solutions.

Reagents	Saliva	Gastric Fluid	Small Intestinal Fluid
NaCl	12.7 mg	310.0 mg	0.2 g
KCl	6.5 g	110.0 mg	25.0 mg
NaHCO_3_	6.2 g	60.0 mg	-
NaSO_4_	4.1 g	-	-
Na_2_HPO_4_	6.5 g	-	-
KSCN	1.5 g	-	-
CaCl_2_·2H_2_O	-	15.0 mg	12.7.0 mg
pH	7.0	3.0	7.0
Distilled water	100 mL	100 mL	100 mL
Others	Urea: 1.8 g; α-amylase: 21.1 mg	Pepsase: 23.6 mg; Gastric lipase: 25.0 mg	Pancreatin: 2.7 g; Bile salt: 3.1 g

pH adjustment with NaHCO_3_ and HCl.

**Table 2:** MW and reducing sugar content of ABP during simulated digestion.

Process	Digestion Time (h)	MW (Da)	Sugar Content (mg/mL)
Saliva digestion	0	41,083 ± 25 ^a^	0.08 ± 0.005 ^a^
	0.5	41,003 ± 16 ^a^	0.10 ± 0.008 ^ab^
	1	40,662 ± 43 ^b^	0.13 ± 0.010 ^bc^
Gastric digestion	0	40,642 ± 40 ^b^	0.13 ± 0.011 ^bc^
	1	40,405 ± 19 ^c^	0.16 ± 0.008 ^cd^
	2	39,592 ± 32 ^d^	0.19 ± 0.015 ^d^
	4	39,562 ± 48 ^d^	0.18 ± 0.010 ^d^
Small intestinal digestion	0	39,511 ± 32 ^d^	0.19 ± 0.009 ^d^
	1	39,538 ± 35 ^d^	0.19 ± 0.012 ^d^
	2	39,525 ± 27 ^d^	0.18 ± 0.015 ^d^
	4	39,573 ± 28 ^d^	0.19 ± 0.011 ^d^

Data are expressed as mean ± SD (*n* = 3). The superscripts in each column indicate the analysis of differences in MW and reducing sugar during simulated gastrointestinal digestion in vitro. There was no significant difference (*p* > 0.05) in those marked with the same letter, while there was a significant difference (*p* < 0.05) in those marked with different letters.

**Table 3:** MW and residual carbohydrate of ABP at different fermentation times.

Fermentation Time	MW (Da)	Residual Carbohydrate (%)	
		ABP	FOS
0 h	39,573 ± 32 ^a^	100 ^a^	100 ^a^
6 h	20,378 ± 31 ^b^	71.8 ± 2.3 ^b^	69.8 ± 1.6 ^b^
12 h	15,842 ± 25 ^c^	68.4 ± 2.2 ^bc^	65.3 ± 2.2 ^bc^
24 h	12,258 ± 22 ^d^	66.3 ± 2.7 ^bc^	61.3 ± 2.5 ^c^
48 h	10,983 ± 24 ^e^	63.1 ± 1.5 ^c^	54.1 ± 6.2 ^d^

Data are expressed as mean ± SD (*n* = 3). The superscripts in each column indicate the analysis of differences in MW and residual carbohydrate of ABP at different fermentation times. There was no significant difference (*p* > 0.05) in those marked with the same letter, while there was a significant difference (*p* < 0.05) in those marked with different letters.

## Data Availability

The original contributions presented in this study are included in the article/Supplementary Materials. Further inquiries can be directed to the corresponding authors.

## Supplementary Materials

The following supporting information can be downloaded at: https://www.mdpi.com/article/10.3390/nu17203249/s1, Figure S1. The rarefaction curves of 16S rRNA in NC mice; Figure S2. The rarefaction curves of 16S rRNA in T2DM mice; Figure S3. The ratio of Firmicutes/Bacteroidetes (F/B) in T2DM mice. ** *p* < 0.01.

## Abbreviations

The following abbreviations are used in this manuscript:

16S rRNA	16S ribosomal RNA
PMP	1-phenyl-3 methyl-5-pyrazolone
ABPF	ABP fermentation samples
T2DM-ABP group	ABP group of T2DM mice
AC	Acarbose
ABP	*Achyrantha bidentata polysaccharide*
AB	Achyranthes bidentata
NC-Blank group	Blank group of NC mice
T2DM-Blank group	Blank group of T2DM mice
FBG	Fasting blood sugar
T2DM-FOS group	FOS group of T2DM mice
FT-IR	Fourier transform infrared spectroscopy
FOS	Fructooligosaccharides
GC-MS	Gas chromatography-mass spectrometry
ABP-G	Gastric-digested ABP
HG-Caco-2	High-glucose Caco-2 cells
HG-CON	HG-Caco-2 cells treated with standard medium
HG-ABPF	HG- Caco-2 cells treated with ABPF medium
HSFD	High-sugar and high-fat diet
MW	Molecular weight
NC	Normal control
NC- Caco-2	Normal Caco-2 cells
NC-CON	NC-Caco-2 cells treated with standard medium
NC-ABPF	NC-Caco-2 cells treated with ABPF medium
NCVP	Nostoc Commune Vauch polysaccharides
PBS	Phosphate buffer saline
PCA	Principal component analysis
PCoA	Principal coordinate analysis
ABP-S	Saliva-digested ABP
SCFAs	Short-chain fatty acids
ABP-I	Small intestinal-digested ABP
TCA	Trichloroacetic acid
T2DM	Type 2 diabetes mellitus

## References

[1] Fu C., Qiu Z., Huang Y., Lin Q., Jin L., Tu H., Ye J., Zheng C., Zhong W., Ma D. (2022). Achyranthes bidentata polysaccharides alleviate endoplasmic reticulum stress in osteoarthritis via lncRNA NEAT1/miR-377-3p pathway. Biomed. Pharmacother..

[2] Lei Y.Y., Ye Y.H., Liu Y., Xu J.L., Zhang C.L., Lyu C.M., Feng C.G., Jiang Y., Yang Y., Ke Y. (2024). Achyranthes bidentata polysaccharides improve cyclophosphamide-induced adverse reactions by regulating the balance of cytokines in helper T cells. Int. J. Biol. Macromol..

[3] Si H., Chen Y., Hu D., Yao S., Yang J., Wen X. (2024). A graminan type fructan from Achyranthes bidentata prevents the kidney injury in diabetic mice by regulating gut microbiota. Carbohydr. Polym..

[4] Zhang M., Wang Y., Zhang Q., Wang C., Zhang D., Wan J.B., Yan C. (2018). UPLC/Q-TOF-MS-based metabolomics study of the anti-osteoporosis effects of Achyranthes bidentata polysaccharides in ovariectomized rats. Int. J. Biol. Macromol..

[5] Xia T., He W., Luo Z.Y., Wang K.X., Tan X.M. (2024). Achyranthes bidentata polysaccharide ameliorates type 2 diabetes mellitus by gut microbiota-derived short-chain fatty acids-induced activation of the GLP-1/GLP-1R/cAMP/PKA/CREB/INS pathway. Int. J. Biol. Macromol..

[6] Li S., Xiong Q., Lai X., Li X., Wan M., Zhang J., Yan Y., Cao M., Lu L., Guan J. (2016). Molecular modification of polysaccharides and resulting bioactivities. Compr. Rev. Food Sci. Food Saf..

[7] Yu Y.H., Wu L.B., Liu X., Zhao L.C., Li L.Q., Jin M.Y., Yu X., Liu F., Li Y., Li L. (2024). In vitro simulated digestion and fermentation characteristics of pectic polysaccharides from fresh passion fruit (*Passiflora edulis* f. Flavicarpa L.) peel. Food Chem..

[8] Li H., Liu S., Liu Y., Li W., Niu A., Ren P., Liu Y., Jiang C., Inam M., Guan L. (2022). Effects of in vitro digestion and fermentation of Nostoc commune Vauch. Polysaccharides on properties and gut microbiota. Carbohydr. Polym..

[9] Wang L., Zeng Z., Lin Y., Zheng B., Zhang Y., Pan L. (2024). In vitro dynamic digestion properties and fecal fermentation of Dictyophora indusiata polysaccharide: Structural characterization and gut microbiota. Int. J. Biol. Macromol..

[10] Fan L., Xia Y., Wang Y., Han D., Liu Y., Li J., Fu J., Wang L., Gan Z., Liu B. (2023). Gut microbiota bridges dietary nutrients and host immunity. Sci. China Life Sci..

[11] Zmora N., Suez J., Elinav E. (2019). You are what you eat: Diet, health and the gut microbiota. Nat. Rev. Gastroenterol. Hepatol..

[12] Zhang D., Liu J., Cheng H., Wang H., Tan Y., Feng W., Peng C. (2022). Interactions between polysaccharides and gut microbiota: A metabolomic and microbial review. Food Res. Int..

[13] Henao-Mejia J., Elinav E., Jin C., Hao L., Mehal W.Z., Strowig T., Thaiss C.A., Kau A.L., Eisenbarth S.C., Jurczak M.J. (2012). Inflammasome-mediated dysbiosis regulates progression of NAFLD and obesity. Nature.

[14] Xu N., Zhou Y., Lu X., Chang Y. (2021). *Auricularia auricula-judae* (Bull.) polysaccharides improve type 2 diabetes in HFD/STZ-induced mice by regulating the AKT/AMPK signaling pathways and the gut microbiota. J. Food Sci..

[15] Di T., Chen G., Sun Y., Ou S., Zeng X., Ye H. (2018). In vitro digestion by saliva, simulated gastric and small intestinal juices and fermentation by human fecal microbiota of sulfated polysaccharides from Gracilaria rubra. J. Funct. Foods.

[16] Chen C., Huang Q., Fu X., Liu R.H. (2016). In vitro fermentation of mulberry fruit polysaccharides by human fecal inocula and impact on microbiota. Food Funct..

[17] Jiao W., Sang Y., Wang X., Wang S. (2023). Effects of 6-Shogaol on glucose uptake and intestinal barrier integrity in caco-2 cells. Foods.

[18] Zhao H., Li M., Liu L., Li D., Zhao L., Wu Z., Zhou M., Jia L., Yang F. (2023). Cordyceps militaris polysaccharide alleviates diabetic symptoms by regulating gut microbiota against TLR4/NF-kappaB pathway. Int. J. Biol. Macromol..

[19] Yang Q., Chang S.L., Tian Y.M., Li W., Ren J.L. (2024). Glucan polysaccharides isolated from Lactarius hatsudake Tanaka mushroom: Structural characterization and in vitro bioactivities. Carbohydr. Polym..

[20] Sun Y., Zhang C., Zhang P., Ai C., Song S. (2022). Digestion characteristics of polysaccharides from Gracilaria lemaneiformis and its interaction with the human gut microbiota. Int. J. Biol. Macromol..

[21] Hu J.L., Nie S.P., Min F.F., Xie M.Y. (2013). Artificial simulated saliva, gastric and intestinal digestion of polysaccharide from the seeds of *Plantago asiatica* L.. Carbohydr. Polym..

[22] Li X., Guo R., Wu X., Liu X., Ai L., Sheng Y., Song Z., Wu Y. (2020). Dynamic digestion of tamarind seed polysaccharide: Indigestibility in gastrointestinal simulations and gut microbiota changes in vitro. Carbohydr. Polym..

[23] Fu C., Ye K., Ma S., Du H., Chen S., Liu D., Ma G., Xiao H. (2023). Simulated gastrointestinal digestion and gut microbiota fermentation of polysaccharides from Agaricus bisporus. Food Chem..

[24] Hays K.E., Pfaffinger J.M., Ryznar R. (2024). The interplay between gut microbiota, short-chain fatty acids, and implications for host health and disease. Gut Microbes.

[25] Yu Y., Shen M., Song Q., Xie J. (2018). Biological activities and pharmaceutical applications of polysaccharide from natural resources: A review. Carbohydr. Polym..

[26] Liu P., Wang Y., Yang G., Zhang Q., Meng L., Xin Y., Jiang X. (2021). The role of short-chain fatty acids in intestinal barrier function, inflammation, oxidative stress, and colonic carcinogenesis. Pharmacol. Res..

[27] Liu X., Cooper D.E., Cluntun A.A., Warmoes M.O., Zhao S., Reid M.A., Liu J., Lund P.J., Lopes M., Garcia B.A. (2018). Acetate production from glucose and coupling to mitochondrial metabolism in mammals. Cell.

[28] Yang X., Zhang M., Liu Y., Wei F., Li X., Feng Y., Jin X., Liu D., Guo Y., Hu Y. (2023). Inulin-enriched Megamonas funiformis ameliorates metabolic dysfunction-associated fatty liver disease by producing propionic acid. npj Biofilms Microbiomes.

[29] Mei X., Li Y., Zhang X., Zhai X., Yang Y., Li Z., Li L. (2024). Maternal phlorizin intake protects offspring from maternal Obesity-Induced metabolic disorders in mice via targeting gut microbiota to activate the SCFA-GPR43 pathway. J. Agric. Food Chem..

[30] Luo J., Liang S., Jin F. (2024). Gut microbiota and healthy longevity. Sci. China Life Sci..

[31] Feng J., Qian Y., Zhou Z., Ertmer S., Vivas E.I., Lan F., Hamilton J.J., Rey F.E., Anantharaman K., Venturelli O.S. (2022). Polysaccharide utilization loci in Bacteroides determine population fitness and community-level interactions. Cell Host Microbe.

[32] Lacombe-Harvey M.E., Brzezinski R., Beaulieu C. (2018). Chitinolytic functions in actinobacteria: Ecology, enzymes, and evolution. Appl. Microbiol. Biotechnol..

[33] Yan H., Fan C.J., Wang Y.J., Liu Z.L., Wang J.Q., Nie S.P. (2025). Structural characterization and in vitro fermentation of the polysaccharide from fruits of Gardenia jasminoides. Int. J. Biol. Macromol..

[34] Fu Q., Tian M., Yang Y., Zhu Y., Zhou H., Tan J., Wang J., Huang Q. (2024). Paotianxiong polysaccharides potential prebiotics: Structural analysis and prebiotic properties. Food Chem..

[35] Chen S., Han P., Zhang Q., Liu P., Liu J., Zhao L., Guo L., Li J. (2023). Lactobacillus brevis alleviates the progress of hepatocellular carcinoma and type 2 diabetes in mice model via interplay of gut microflora, bile acid and NOTCH 1 signaling. Front. Immunol..

[36] Meng F., Zhang F., Meng M., Chen Q., Yang Y., Wang W., Xie H., Li X., Gu W., Yu J. (2023). Effects of the synbiotic composed of mangiferin and Lactobacillus reuteri 1-12 on type 2 diabetes mellitus rats. Front. Microbiol..

[37] Zhu X.X., Zhao C.Y., Meng X.Y., Yu X.Y., Ma L.C., Chen T.X., Chang C., Chen X.Y., Zhang Y., Hou B. (2024). Bacteroides uniformis Ameliorates Carbohydrate and Lipid Metabolism Disorders in Diabetic Mice by Regulating Bile Acid Metabolism via the Gut-Liver Axis. Pharmaceuticals.

[38] Delzenne N.M., Neyrinck A.M., Backhed F., Cani P.D. (2011). Targeting gut microbiota in obesity: Effects of prebiotics and probiotics. Nat. Rev. Endocrinol..

[39] Niu H., Zhou M., Ji A., Zogona D., Wu T., Xu X. (2024). Molecular Mechanism of Pasteurized Akkermansia muciniphila in Alleviating Type 2 Diabetes Symptoms. J. Agric. Food Chem..

[40] Dong L., Xu Z., Huang G., Zhang R., Deng M., Huang F., Su D. (2023). Lychee Pulp-Derived dietary Fiber-Bound phenolic complex upregulates the SCFAs-GPRs-ENS pathway and aquaporins in Loperamide-Induced constipated mice by reshaping gut microbiome. J. Agric. Food Chem..

[41] Xue H., Liang B., Wang Y., Gao H., Fang S., Xie K., Tan J. (2024). The regulatory effect of polysaccharides on the gut microbiota and their effect on human health: A review. Int. J. Biol. Macromol..

