# Supplementary material for: The Anti-Digestive Characteristics, Effects of Prebiotic Properties on NC and T2DM Mice of Achyranthes bidentata Polysaccharide, and the Hypoglycemic Effect of Its Fermentation Products
Source: Nutrients. 2025 Oct 16;17(20):3249. doi: 10.3390/nu17203249 (PMC12567556; doi:10.3390/nu17203249)
Supplement: Supplementary file 1 [file nutrients-17-03249-s001.zip › supplementary materials.pdf]

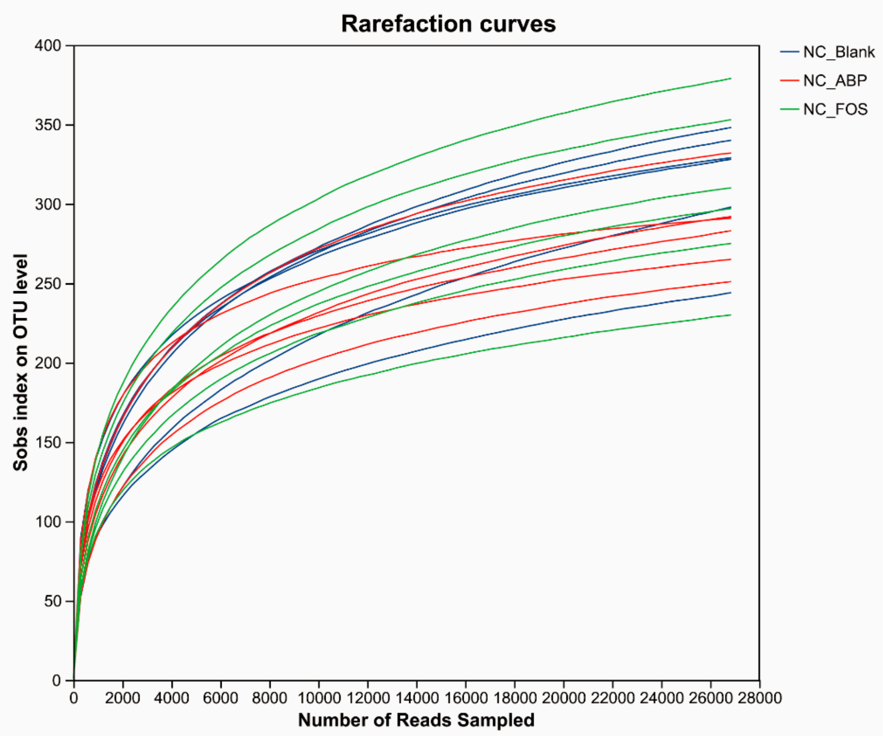

Figure S1. The rarefaction curves of 16S rRNA in NC mice.

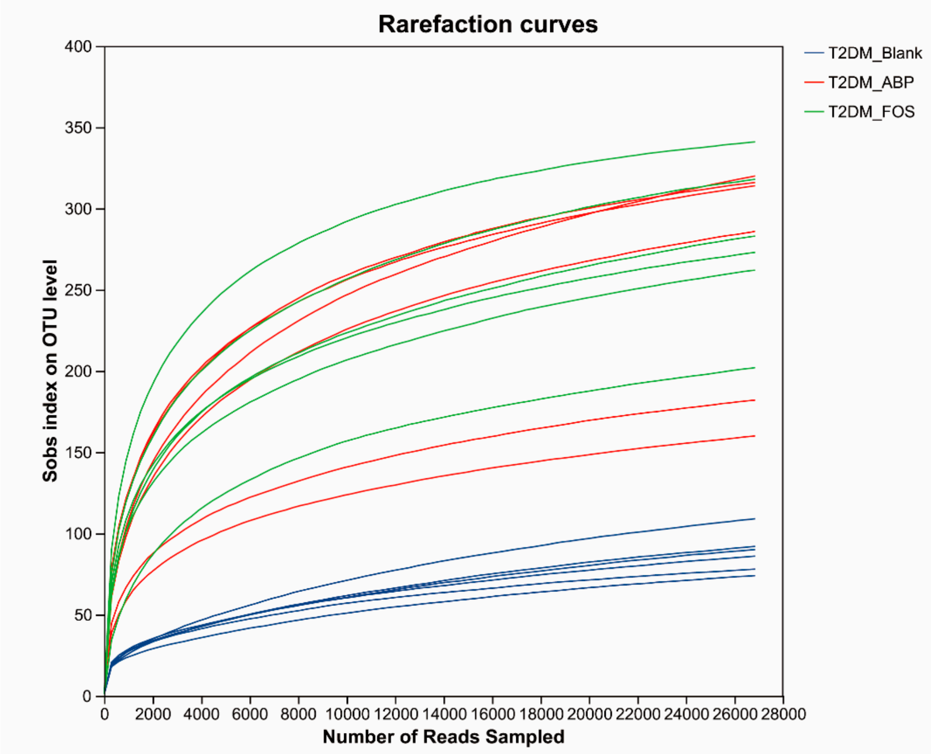

Figure S2. The rarefaction curves of 16S rRNA in T2DM mice.

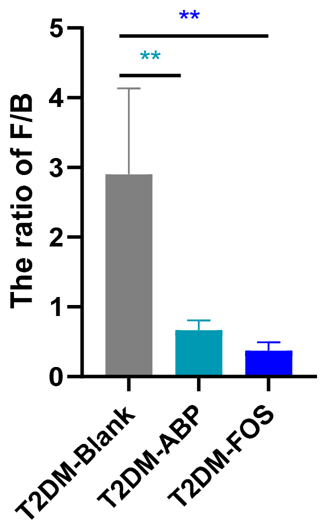

Figure S3. The ratio of Firmicutes/Bacteroidetes (F/B) in T2DM mice. $^{**}P<0.01$.
